# A mixed-method study exploring experiences, perceptions, and acceptability of using a safe delivery mHealth application in two district hospitals in Rwanda

**DOI:** 10.1186/s12912-022-00951-w

**Published:** 2022-07-04

**Authors:** Aurore Nishimwe, Daphney Nozizwe Conco, Marc Nyssen, Latifat Ibisomi

**Affiliations:** 1 School of Public Health, Faculty of Health Sciences, University of the Witwatersrand, 1 Smuts Avenue, 2000 Braamfontein, South Africa; 2 School of Public Health, College of Medicine and Health Sciences, University of Rwanda, P.O. Box 3286, Kigali, Rwanda; 3 Department of Biomedical Statistics and Informatics, Vrije Universiteit Brussel, Brussels, Belgium; 4 Nigerian Institute of Medical Research, 6 Edmund Cres, Yaba, Lagos Nigeria

**Keywords:** BEmONC, mHealth, Mixed-methods, Nurses and midwives, Safe delivery application, Rwanda

## Abstract

**Background:**

Innovative use of mobile health (mHealth) technology in timely management of childbirth complications is a promising strategy, but its evidence base is limited. The Safe Delivery mHealth Application (SDA) is one of the recent mhealth applications (loaded in smartphones) which is a clinical decision support and training tool for basic emergency obstetric and newborn care (BEmONC). This paper describes, the health providers’ experiences, perceptions, and acceptability of using the SDA, as well as the perceptions of key stakeholders.

**Methods:**

A mixed-methods approach was utilized. Quantitative methods consisted of a self-reported acceptability survey, administered to 54 nurses and midwives, including questions on their usage and perceptions of the SDA. Descriptive statistics were employed to analyze the survey data. Qualitative methods included two focus group discussions with 24 nurses and midwives, and six key informant interviews with stakeholders (maternity matrons, responsible for maternal and child health, and district hospital managers). Thematic analysis was performed and selected quotations used to illustrate themes. The study took place in two district hospitals in Rwanda.

**Results:**

Quantitative results found that 31 (57.4%) participants used the SDA four to six times per week. Many participants felt more confident (53.7%) and better at their job (40.7%) since having the SDA. Likert scale survey responses (1–5, 1 = Strongly Disagree, 5 = Strongly Agree) indicated general agreement that SDA is easy to use (Mean = 4.46), is an effective decision support tool (4.63), and training tool (4.65). Qualitative results included themes on perceived usefulness; professional growth acquired through the use of the SDA; SDA, an empowering, intuitive, and user-friendly technology; desired SDA features and functions; benefits of SDA as perceived by key informants, and future use of the SDA.

**Conclusions:**

The nurses and midwives perceive the SDA as having improved their ability to manage childbirth complications. Key stakeholders also perceive the SDA as a useful tool with a reasonable cost and recommend its implementation in routine practices. This study deepens the understanding of the potential benefits of mHealth such as the SDA in low-income settings, like Rwanda. It also provides more evidence on the impact of mHealth in assuring quality BEmONC.

**Supplementary Information:**

The online version contains supplementary material available at 10.1186/s12912-022-00951-w.

## Background

Globally, maternal and newborn deaths remain a public health concern despite several efforts that have been/are being made to reduce the deaths. It is estimated that 295,000 maternal deaths, 2 million stillbirths and 2.5 million early newborn deaths occur annually in the world [1, 2]. More than 90% of these deaths happen in low- and middle-income countries, like Rwanda [3, 4]. Most of these deaths are attributable to preventable causes such as post-partum haemorrhage (PPH), newborn asphyxia, pre-eclampsia, obstructed labor, infections, and hypertensive disorders [5]. The WHO, UNICEF, and UNFPA have identified essential interventions known as Basic Emergency Obstetric and Newborn Care (BEmONC) for prevention and treatment of the preventable causes of maternal and newborn deaths [6, 7]. The BEmONC package includes the management of PPH, the removal of a retained placenta, the management of maternal sepsis, the management of hypertensive disorders, the management of prolonged labor, the management of complications of abortion, and neonatal resuscitation (NR) [6]. These services can be provided by nurses and midwives. There is evidence that BEmONC is a key intervention to reduce death and disability of mothers and newborns [7]. However, the available literature remains characterized by gaps in assuring the quality of BEmONC.

The quality of BEmONC services is important to improving maternal and newborn health [8]. The research evidence suggests that barriers to instituting quality BEmONC are complex and are often linked to limited knowledge and skills among health care providers, lack of access and adherence to evidence-based clinical guidelines, insufficient monitoring to inform appropriate responses, and lack of some essential materials [3]. Besides, research recommends the use of mobile health (mHealth) to improve health systems, as a solution that could potentially support in addressing some of the health systems challenges and improve service delivery [9, 10].

The safe delivery mHealth application (SDA) is a novel mHealth application which is a decision support and training tool for BEmONC that was developed by the Maternity Foundation [11]. The main purpose of the SDA is to make a healthcare provider better prepared to prevent, detect, and manage life threatening complications to mothers and newborn via offering evidence-based BEmONC [11]. The SDA contains animated videos with instructions on management of PPH and NR. The SDA offers the opportunity to watch the entire instructional animated video (8–15 min long) as well as choosing to see a specific procedural step of each video (1–3 min long). Besides, the SDA contains a catalogue of action cards with essential recommendations to support clinical decision making in an emergency situation when the user does not have time to review a video. The SDA also include a comprehensive drug list which contains useful information on the various essential drugs needed to perform PPH management and NR. Further, the SDA describes certain essential procedures step-by-step and required equipments. In addition, the SDA has a self-explanatory learning platform ‘My Learning’ that provides learning and assessment strategies based on key elements within the clinical content found in the SDA. To stimulate use, the SDA sends weekly notifications with quiz questions and a direct link to the films where the information to answer the question is found. The clinical content of the SDA was developed following international WHO guidelines but focusing on key essential lifesaving interventions of BEmONC [12].

The SDA can be downloaded free of charge for iPhone at: https://itunes.apple.com/dk/app/safe-delivery/id985603707?mt=8 and for Android at: https://play.google.com/store/apps/details?id=dk.maternity.safedelivery&hl=en.

The present paper, was derived from a broader study about the implementation of the SDA in handling two elements of the BEmONC: PPH and NR in two district hospitals in Rwanda. Such focus was informed by evidence that indicated PPH and newborn asphyxia as the priority challenges in Rwanda [13, 14]. In 2019, Rwanda’s maternal mortality was estimated at 203 deaths per 100,000 live births and its neonatal mortality rate was 19 deaths per 1000 live births [15] despite 91% of births taking place in healthcare facilities and attended by healthcare professionals [16]. Therefore, more efforts are still needed to reduce the numbers of deaths. The SDA was piloted in two district hospitals in Rwanda. This pilot implementation process was divided into three phases (pre-intervention, intervention, and post-intervention). The first phase (pre-intervention) involved the presentation of the research to different stakeholders and the baseline study (surveys, records review, and focus group discussions). The second phase (intervention) included the capacity building in the usage of the SDA among nurses and midwives; the SDA provision and piloting; and the SDA implementation for a period of six months. The third phase (post-intervention) included the implementation evaluation and encompassed the endline study (surveys, records reviews, focus group discussions, and key informant interviews). More details are provided in the protocol paper for this research [17]. The present paper explores the end-users’ experiences, perceptions and acceptability of the SDA and key stakeholders’ perspectives.

## Methods

### Study design

This paper describes part of a larger study about the implementation of the SDA in BEmONC services in two district hospitals in Rwanda. The larger study methodology and the evaluation of initial outcomes have been reported elsewhere [17–20]. The part of the study presented in this paper, used mixed research methods to explore end-users’ experiences, perceptions, and acceptability, and explore the key stakeholders’ perceptions of the SDA. Mixed-methods is defined as a research design that involve the combination of quantitative and qualitative research approaches in order to obtain a comprehensive understanding of the research question [21, 22].

### Study setting

The study took place in two district hospitals in Rwanda: Masaka hospital in Kigali, an urban province; and Nyamata hospital located in the eastern rural province [23].The two hospitals were selected out of 12 district hospitals in the two provinces because both had a high number of deliveries per year [24] and had been offering BEmONC services for more than five years.

### Study participants

 The study adopted a purposive sampling and included 60 participants, the nurses and midwives (*n* = 54) and stakeholders (*n* = 6). Nurses and midwives who deal with childbirth in the maternity departments of the two district hospitals were introduced to the study and invited to participate in the study during the face-to-face morning staff meetings. The inclusion criteria were as follows: having work experience of at least six months in obstetric care; being full-time employed in the selected hospitals; and willing to participate in the study. The selected stakeholders (key informants in the management position at the district hospital level) were approached individually and consented to participate in the study. They included maternity matrons (*n* = 2), managers responsible for maternal and child health (*n* = 2), and district hospital directors (*n* = 2).

### Data collection and analysis

#### Quantitative data

The quantitative part of the research entailed asking all nurses and midwives who were part of implementation of the SDA to complete a self-administered questionnaire (Additional file 1) evaluating usage of the SDA, as well as their perception and acceptability. A pilot test of the self-administered questionnaire was conducted with 2 nurses and 3 midwives in a different district hospital prior to the survey. The survey was conducted at endpoint once users had used the SDA for six months. Responses were analysed descriptively using Stata version 16 (StataCorp LLC).

#### Qualitative data

Two methods were used to collect qualitative data - focus group discussions (FGDs) and key informant interviews (KIIs).

Focus groups discussions explored in depth the participants’ experiences and views on the functionality of the SDA. Two FGDs were conducted, one in each of the study hospitals. In each hospital, 12 participants were recruited and total number of FGD participants is 24. The participants included in the FGDs were selected based on their interest and availability. We sought perspective diversity in terms of age, gender, and experience [25] and these criteria were considered in both groups.

The Key Informant Interviews were conducted with six participants (three from each hospital), who were selected because of their leadership/management portfolios in the participating hospitals. The purpose was to get their views on the feasibility of adopting the SDA for wider implementation with the focus on its costs, possible scalability and sustainability. The interviews with key stakeholders started with an introduction of the SDA to the key informant including an explanation of the features and modules of the SDA, and a demonstration of how the SDA functions using a smartphone. After the introduction of the SDA, the key informant was invited to ask questions and comment on the SDA. Once their concerns were addressed, the researcher presented the estimated total cost that was used for piloting the SDA in two district hospitals (Masaka and Nyamata) in Rwanda. The estimated total cost was 10.076.410 Rwf ($10,000) including the technology cost = 4.504.000 Rwf ($4470.56), the training cost = 2.083.410 Rwf ($2067.94) and the intervention cost = 3.489.000 Rwf ($3463.09). Then, participants were asked to provide their perceptions of the SDA and their opinions on its cost, scalability, and sustainability.

The interview guides (Additional file 2) were developed in English, translated to Kinyarwanda, and translated back to English in a blinded manner to ensure accuracy and equivalence [26]. To ensure the quality and adequacy of the interview guides, they were pretested with two participants at a different district hospital. The interviews were conducted face to face in March 2020 and were held in private rooms at both Masaka and Nyamata district hospitals to ensure confidentiality. Interviews were conducted in the language of the participants’ preference, which was Kinyarwanda. The first author conducted the interviews with assistance of an experienced research assistant who assisted in taking notes and supervising the tape recording. FGDs lasted between 60 and 0 min and KII interviews between 30 and 45 min. All FGDs and KIIs were digitally recorded and transcribed in Kinyarwanda by two research assistants. Transcriptions were done following the true verbatim method to accurately capture meanings, perceptions, and context [27]. Then, transcripts were translated from Kinyarwanda to English by a professional translator. In order to ensure that everything was translated as correctly as possible, all transcriptions were compared with the audio files and field notes. The data were analyzed using thematic analysis with a hybrid approach (inductive and deductive) [26]. Two authors (AN and DNC) first read all transcripts independently, then jointly developed a codebook. Disagreements were resolved by discussion. The final list of codes was applied to all transcripts using Nvivo 11 Plus software. Coded output was then read, merged into major themes, and illustrative quotations were identified. The consolidated criteria for reporting qualitative research [28] guided reporting for the qualitative data (Additional file 3).

Findings from both quantitative and qualitative data were integrated during the data-interpretation stage. The goal was to obtain different but complementary data that validate the overall results about the SDA acceptability.

### Ethical considerations

Ethical approvals were obtained from the Human Research Ethics Committee of the University of the Witwatersrand (M190258) and the University of Rwanda, College of Medicine and Health Sciences’ Institutional Review Board (No. 377/CMHS IRB/2018). All participants gave written informed consent and consents for recording the interviews prior to their participation. Participants were assured that their identities would remain anonymous in all reporting of the study and that their personal information would be kept confidential. All methods in this study were carried out in accordance with relevant guidelines and regulations in the Ethical Declarations.

## Results

### Characteristics of quantitative study participants

A total of 54 nurses and midwives completed the acceptability survey. The majority of participants were midwives (*n* = 33). More than half were from Masaka district hospital (*n* = 30, 56%), the majority were female (*n* = 33, 61%), their average age was 33 years (SD = 7.1). The level of education was predominantly the advanced diploma (A1) in midwifery with 27 of 54 (50%) participants having advanced diploma (A1 level) in midwifery. The least represented level of education was the secondary school level (A2) in nursing with only one participant with A2 level in nursing. A detailed description of the survey respondents is provided in Table 1.

**Table 1:** Demographic characteristics of the acceptability survey respondents (*N* = 54)

			n (%)
**Hospital Affiliation**			
Masaka District Hospital			30 (55.6)
Nyamata District Hospital			24 (44.4)
**Sex**			
Male			21 (38.9)
Female			33 (61.1)
**Education Level**			
Midwife A0			6 (11.1)
Midwife A1			27 (50)
Nurse A0			5 (9.3)
Nurse A1			15 (27.8)
Nurse A2			1 (1.8)
**Age, years, Mean (SD)**			33 (7.12)

*Abbreviations:* % Weighted percent, *A0 *bachelor’s degree, A1 Advanced Diploma, *A2 *Secondary school level

### The nurses’ and midwives’ experiences of the SDA during the study implementation

#### The utilization of the SDA by nurses and midwives

The majority of participants (*n* = 31) reported using the SDA four to six times per week. Features of the SDA that were most consulted include; the action cards (*n* = 15), my learning (*n* = 14) and the drug list (*n* = 10). A half of the participants used the SDA to revise their knowledge. Other participants used the SDA in emergency situations (*n* = 14), in a normal work situation (*n* = 5) and to discuss with co-workers (*n* = 8). Nurses and midwives predominantly accessed the SDA on both their own smartphones and on the smartphones provided by the researcher (*n* = 28). A large number of participants (*n* = 35) often used the SDA with a co-worker. Participants reported having access to most of the equipments and drugs seen in the SDA (*n* = 40). More details on the SDA use and evaluation by end-users are shown in Table 2.

**Table 2:** SDA access and evaluation by nurses and midwives (*N* = 54)

			n (%)
**Frequency of SDA use in a week**			
I used it almost every day			15 (27.8)
I used it 4–6 times			31 (57.4)
I used it 1–3 times			8 (14.8)
I didn’t use it at all			0 (0)
**Features of the SDA most consulted**			
Videos			9 (16.7)
Action cards			15 (27.8)
Drug list			10 (18.5)
Procedures			6 (11.1)
My Learning			14 (25.9)
**Most often quoted reasons for using the SDA**			
To revise my knowledge			27 (50)
During an emergency			14 (25.9)
In a normal work situation			5 (9.3)
To discuss with co-workers			8 (14.8)
**The device often used to access the SDA**			
Own phone			7 (13)
The phone provided by the researcher			19 (35.2)
Both			28 (51.8)
**With who do you often use the SDA?**			
By myself			19 (35.2)
With a co-worker			35 (64.8)
**Access to the equipment and/or drugs seen in the SDA**			
All			14 (25.9)
Most			40 (74.1)
None			0 (0)

*Abbreviations:* *Wt.% *Weighted percent, *SDA *Safe Delivery mHealth Application

When rating their experiences of using the SDA particularly, half of respondents (*n* = 27) indicated that it was very easy to the SDA, however one participant reported that it was very difficult to use a smartphone. SDA features that were most appreciated included: the action cards (33%), the drug list (22%), and my learning (19%). They also reported feeling more confident (53.7%) and becoming better (40.7%) at doing their jobs at work. Few participants (*n* = 5) reported what they liked least about the SDA, three participants stated that they did not understand instructions, and the other two said that it was difficult to navigate the smartphone. More details on the experiences with the SDA and its added value are displayed in Table 3.

**Table 3:** Experiences with the SDA and its added value (*N* = 54)

			n (%)
**Experience with the smartphone and the SDA**			
Very easy			27 (50)
Somewhat easy			23 (42.6)
Somewhat difficult			3 (5.6)
Very difficult			1 (1.8)
**Features of the SDA most appreciated**			
Action cards			18 (33.3)
Videos			10 (18.5)
Drug list			5 (9.3)
Procedures			12 (22.2)
My Learning			9 (16.7)
**Added value from the SDA**			
I have become better at doing my job.			22 (40.7)
I feel more confident at work.			29 (53.7)
Fewer mortalities/fatal cases			3 (5.6)
I did not notice any change.			0 (0)
**What do you like least about the SDA?**			
I do not understand the instructions.			3 (5.6)
I think it is difficult to navigate the smartphone.			2 (3.7)
The app does not add to my existing knowledge.			0 (0)
None applicable.			49 (90.7)

*Abbreviations:* *Wt.% *Weighted percent, *SDA *Safe Delivery mHealth Application

### Acceptability of the SDA as rated by nurses and midwives

Nurses and midwives were asked to rate their acceptance of the SDA in a Likert scale. They were asked whether they “strongly disagree”, “disagree”, were “undecided”, “agree”, or “strongly agree” to a series of statements relating to the SDA acceptability. Responses were assigned point values as follows; strongly disagree = 1, disagree = 2, undecided = 3, agree = 4, strongly agree = 5. Likert scale survey results showed that the SDA was: well understood (Mean = 4.00); easy (4.46) and comfortable (4.31) to use; an effective decision support (4.63) and training (4.65) tool; and useful in improving efficiency in BEmONC (4.59). Most participants reported that they intended to continue using the SDA in daily practices (4.68). On the other hand, a lower rating was given to using the SDA when seeing notifications (2.48), implying that most participants did not only use the SDA when they saw notifications. The notifications are push messages that appear occasionally on the phones of the SDA users to remind them to revisit the SDA. The messages mainly guide the users to interesting parts that they may want to explore in the SDA. Overall participants reported good acceptability of the SDA. Likert scale responses are highlighted in Table 4.

**Table 4:** Likert scale survey responses of nurses and midwives about SDA acceptance

Statement	N	Mean (SD) [Range]
I have a good understanding of what the SDA is	54	4.00 (0.71) [3-5]
The SDA is easy to use	54	4.46 (0.50) [4-5]
I am comfortable using the SDA	54	4.31 (0.57) [4-5]
The SDA is an effective decision support tool for BEmONC	54	4.63 (0.69) [4-5]
The SDA is an effective training tool for BEmONC	54	4.65 (0.48) [4-5]
The SDA is useful in improving efficiency in BEmONC	54	4.59 (0.56) [3-5]
I intend to continue using the SDA in my daily practices	54	4.68 (0.50) [4-5]
I use the SDA when I see notifications	54	2.48 (0.40) [1-3]

Likert scale response options were as follows: 1Strongly Disagree, 2Disagree, 3Neutral, 4Agree, and 5Strongly Agree

### Perceptions about the usefulness and acceptability of the SDA

**Fig. 1:**
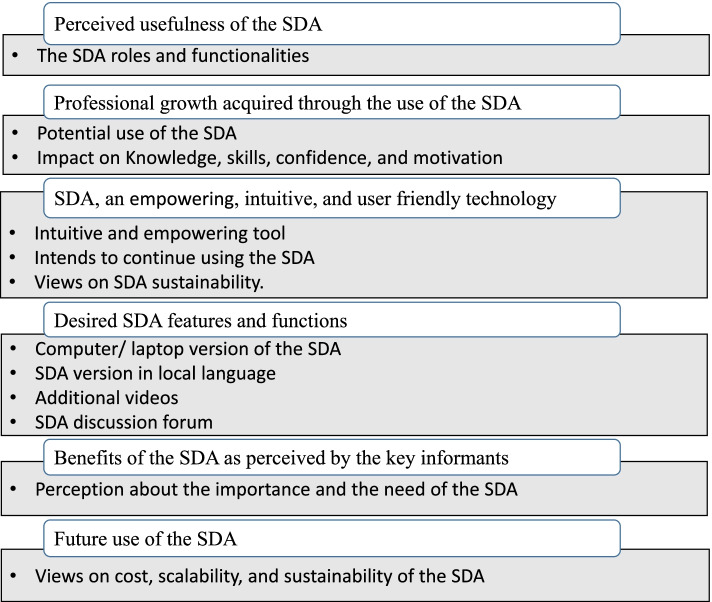
Thematic framework

The results of the interviews with end-users of the SDA emerged into four main themes: (1) the perceived usefulness of the SDA; (2) professional growth acquired through the use of the SDA; (3) SDA, an empowering, intuitive and user-friendly technology; and (4) the desired SDA features and functions. The results of the interviews with key stakeholders revealed two main themes (1) benefits of the SDA as perceived by the key informants, and (2) future use of the SDA. These identified themes are shown in Fig. 1. More details on the main themes and sub-themes are presented in the discussion of results, and are illustrated by verbatim quotations from the two FGDs and six KIIs.

### Interviews with end-users of the SDA

#### Perceived usefulness of the SDA

Participants described the SDA as a useful, intuitive, empowering and practical tool that supported their daily practices. They described how it provided important information and guidelines on management of obstetric emergencies, and thus, increases their knowledge and skills on how to manage normal childbirth as well as birth complications.

*“This application came as a golden opportunity for us …normally, it is very hard to go to a library all the time to read; but the library, I am hereby talking about is that SDA application we have received…It provides very useful information and guidelines that upgrade our knowledge and skills in delivery care” *Midwife, FGD1.

In addition, nurses and midwives reported using the SDA both as a decision support tool while managing birth complications including PPH or NR, and as a debriefing tool for reflections on performed care to identify if essential procedures had been missed.

*“When you have an emergency case to manage, you don’t have difficulties figuring out how to handle it…all the steps are clearly outlined in this application and so well summarized that you immediately find what you need whenever you refer to it…The SDA support our clinical decisions in emergency situations…Sometimes, when I go home from a night duty, on the bus, I consult the SDA to see if I have performed well during the night” *Nurse, FGD2.

Participants also described the SDA as a practical and easy to use tool that helps strengthen their theoretical knowledge. They articulated how the SDA helped them remember the management of rare clinical cases. Procedures for those rare cases are easy to forget as they rarely had to apply the theory learnt from school into practice.

*“Before SDA, you could sometimes start wondering what management was appropriate for a case which is not so frequent after you had forgotten…Now, we refer to the SDA when there is a doubt about anything… With this new application, we have become all fresh and we can easily remember the essential procedures step by step as per international standards” *Midwife, FGD1.

Other participants expressed appreciation of the SDA as a training tool, highlighting the ingenuity of the visuals when dealing with life threatening complications of mothers and their newborns. They considered the SDA to be a kind of simulated clinical training that continuously enhances their knowledge and practical skills, contributing to their professional growth. They also reported improved performance when managing childbirth complications. Such improvement was attributed to continuous learning, facilitated by the SDA and the fact that learning could take place anywhere.

*This application has been an important learning tool…I consider it as a training manual with all clinical guidelines accompanied by video materials… we learn a lot from the SDA in addition to what we learned at school…You can learn from this application from anywhere: at the hospital, on the bus, at home or anywhere. So, having your telephone means being able to learn more anywhere.” *Midwife, FGD2.

Then again, participants revealed that they used to consult other internet sources for learning like youtube. They found such sources not so easy to use, as it was hard to get concise updated information and guidance in a timely manner, the way the SDA does.

*“We used to visit youtube to learn something about the services we deliver, but most materials in there are not so clear as in this app. When you consult the SDA, you find that everything is easy to understand. You quickly get information on which medication is good for which case…” *Midwife, FGD1.

*“Some of us are mentors in health centers and we have been consulting this application while preparing training materials” *Midwife, FGD1.

Some found the SDA notifications useful as reminders for them to continue learning. The notifications that appeared intermittently on their phones, reminded them to revisit the SDA that also pointed them to other interesting parts of the SDA that they may want to explore.

*“We have posters displayed in maternity telling us what to always bear in mind. Some of the information on them is “remember to close the door to keep the newborn warm” … I compare this with the SDA notifications that I frequently get on my smartphone…these are very nice reminders that I have to keep learning” *Midwife, FGD2.

*“I would come back to what a colleague said about SDA notifications. This is a way of alerting you and I like that… There exists a wrong belief that nurses don’t like reading. Maybe, it is true but this application is not too demanding. It doesn’t consume your time as if you go to a library… This app is a good source and we need to regularly use it” *Nurse, FGD1.

#### Professional growth acquired through the use of the SDA

The perceived usefulness of the SDA described by the participants was associated with substantial improvements in their childbirth care practices. Nurses and midwives narrated experiencing an increase in their knowledge, skills, and confidence after using the SDA and how such professional growth has been reflected in the quality service they offer.

*“The reason number one I like this app is that it upgrades my knowledge and skills, and makes me confident than before…it is a kind of continuous professional development…It is so good in a way that if you fail to understand the text when reading, you have an option to search for more explanation using videos. It is really very useful…” *Nurse, FGD2.

*“The biggest difference for me when I compare now and the past experience is service delivery which has so positively improved. The special aspect of the application is that the content therein has everything we theoretically studied at school” *Midwife, FGD2.

The study participants indicated that the SDA has helped them to recollect and revise their skills, which have made them more capable at managing childbirth complications in accordance with the recommended essential procedures. One of the significant changes expressed by participants was that they had learned the lifesaving steps of how to stop a bleeding by applying bimanual compression.

*“This application has been helping us refresh our memories and equipping us with techniques that we use to handle obstetric emergencies.… My colleagues have talked about bimanual compression…You may even not know anything about it … Then, when you try to familiarize with it on the SDA, you get to know about it and perform it correctly” *Midwife, FGD1.

Other participants explained how the SDA supported them during practice. They described the before SDA childbirth care experience and compared it with after SDA experience by highlighting the specific instances in which the SDA supported them. Previously, in situations where nurses and midwives were struggling to assist a mother or a newborn, they would immediately call upon a medical doctor to come and help. And, sometimes the doctor assigned to maternity department was busy with caesarean surgery which would delay his assistance to attend to the complicated case. But, now with the support of the SDA, nurses and midwives have been able to provide essential assistance to mothers and newborns while waiting for the doctors.

*“I agree that BEmONC services were also delivered before we started using SDA but the situation was not the same. For example, when there was a complication with a patient, the only thing we would do was to ask a colleague or to call a medical doctor. This was time consuming and would delay service delivery and could complicate the situation even cause maternal deaths... However, when there is a problem nowadays, you just take your phone, go to the application and get a quick solution. As a result, our patients get quick and more efficient service” *Midwife, FGD2.

Both nurses and midwives revealed that they used to fear occurrence of birth complications, because of the limited skills and experience on how to manage such events. They noted that the SDA had reduced this fear and had led them to have more confidence in their work.

*“In using this app, you are always aware of what might happen in a given case. So, when you have a real case, you don’t panic much…For instance, when it comes to PPH management, with the SDA you can easily figure out the steps to follow*” Nurse, FGD1.

*“This application can help you when you are stressed. You may have had a lot of cases to manage in a day and end up in confusion somewhere. When this happens, you take your phone and see what should be done to handle a particular case with confidence.” *Midwife, FGD2.

Some participants described how solving problems using the SDA motivated and enabled them to be more efficient in providing care at work.

*“when you have a problem and you find a way to solve it, that is self-explanatory; you feel motivated automatically. As we get support from the application, our job becomes easier and more efficient” *Midwife, FGD1.

Further, nurses and midwives explained that they used the SDA either at home to update knowledge or while they were at work, either before a delivery when the woman is in labor or when complication arises in the course of a delivery. Some participants described using the SDA to guide co-workers when they work in pairs in order to follow the necessary steps during a delivery. In emergency situations, the SDA was often used as a decision support tool to see how to manage e.g. PPH and neonatal resuscitation or as a reminder of drug dosages and administration. Participants also acknowledged having access to most of the equipments and drugs seen in the SDA.

*“We have most of the materials we need to perform well. But, the SDA has been there to support us in upgrading our knowledge wherever we are - at home, before assisting a delivery or during an emergency…You may forget something like medication dosage, even though you learned it at school… but since we got this app, it helps us to remember the dosages in emergencies, we use it as a job aid in a simple way” *Nurse, FGD2.

### SDA an empowering, intuitive and user friendly technology

The study participants also voiced that the SDA is easy to use, intuitive, user friendly, and enabled them to provide first aid to patients before calling upon doctors.

*This application is special in that it is so focused and shows you clear sequences…For example, you can easily find what specifically refers to steps for removing a retained placenta when you have such a case. We really like it because it is easy to use” *Midwife, FGD2.

*“I would say something additional. With this application, we have learnt how at least one can provide first aid for a mother or do resuscitation for a newborn… That is so important because you don’t need to call upon a doctor for everything” *Midwife, FGD2.

Likewise, participants indicated that the SDA has helped them to get a common understanding with medical doctors about managing birth complications. They explained on how consulting the SDA helped them to have informed recommendations and they were happy to contribute with confidence to care plans and sometimes supporting doctors.

*“I would say that when I have a special case, I first consult the application, see how I can myself manage it before I talk to a medical doctor. Even when I have to talk to the doctor, at least I provide an informed suggestion and contribute to further treatment steps. We are supporting our doctors much more.” *Midwife, FGD1.

Then again, nurses and midwives described how the SDA has helped them to get updated information and to have a common understanding among themselves during practice.

*“I can easily remember what I studied in school and when I tell my colleague X, he sometimes tells me a different story. This is due to the fact that he studied so recently, let’s say three years ago, while I studied the similar subject 12 years ago…There is a need for all of us to have the same updates. This application is a solution because it keeps getting updated” *Nurse, FGD1.

When asked if they will continue using the SDA in their future practice. Both nurses and midwives stated that due to the SDA benefits, it is their own advantage to continue using it in their routine practice for efficient provision of care.

*“I would say that we have been lucky to be the champions of this application. I agree that we have a lot of work to do in maternity but we can’t forget that we also need updates in our career…We will always need to refer to the app in our routine practices and find some time to learn new things from the SDA. We shall keep using it and it is for our own interest” *Midwife, FGD2.

*“This application is user friendly, affordable, covers a lot of areas, and does not take your internet megabytes when using… It is not like a book or a computer that would be heavy for you to carry…It is in a mobile phone that you always carry in your hands…You can check up anything, anywhere and anytime.… Unless something unexpected happens, I don’t see how people can stop using the SDA” *Midwife, FGD1.

When asked how they perceive the SDA sustainability in district hospitals in Rwanda, all nurses and midwives indicated that the SDA will be likely sustained.

*“This application will be maintained because it is an important tool for us to deliver good service to the patients. When you use it properly, you deal with various cases with confidence and you perform your duties with success” *Midwife, FGD1.

*“I am also confident that SDA will be sustained for two main reasons. The first reason is that it is easy to use and provides text content accompanied by video materials. Secondly, SDA is so relevant in that it is equipped with modules that reflect our day to day clinical practices” *Midwife, FGD2.

Furthermore, participants requested the SDA scalability to the whole country of Rwanda in consideration of the present country’s agenda to support the adoption of technology in healthcare service delivery.

*“Personally, I would say that this is the most appealing application on my phone…When we are discussing with colleagues on social media (WhatsApp), there may be some questions raised in relation to our career. When there is a confusion, I immediately take my phone, get reliable information from the SDA and in a fast way I share with them… Is it possible to implement this app in all hospitals in Rwanda? …Since most people have smartphones, this should not be a problem, also our country is currently promoting application of technology in healthcare. I think this would make a lot of difference” *Midwife, FGD1.

*“I have to tell you that this is a very good application, those using the application have already started seeing the benefits and they gain a lot from it. I wish it could have been shared with other hospitals too. It should be scaled up to national level. I wish to see all midwives in Rwanda using this app… It is easy to use, once you have it installed, you don’t need much guidance on how to use it, then we will be able to save more maternal and newborn lives” *Midwife, FGD2.

*“I remember I was in Musanze some time back and when I talked about this application to my friends, they were surprised. They started asking me how they can access it too. I just showed them and they became very interested. I am inviting everyone to have this spirit of sharing so that it reaches more people” *Nurse, FGD1.

Other participants recommended that the SDA should be part of learning materials used at University.

*“Ever since I started using the SDA, there has been no single case that I have failed to get information about in this application. I think this application should be part of tools that midwifery students use at University before they graduate. If this is done, using it at workplace would be easier. I am confident that if you once saw something at school and you need to use it in your job, it becomes easily implementable” *Midwife, FGD1.

### Desired SDA features and functions

The study participants were asked if there might be any other information or functionalities they would want to be added to the SDA for improving the application. Most participants stated that the SDA is useful as it is. However, some participants suggested features and functions they wish to be added to the SDA. Firstly, participants requested if the SDA could have a version for desktop and laptop computers so that it could be installed in computers available at their respective hospitals or if they could access the SDA using a desktop/laptop browser.

*“I wonder if this application can’t be installed in our office computers because sometimes one’s phone might not be available maybe because it is not charged, it is stolen or broken or the owner simply doesn’t have it with him/her. Having the application available in various devices would be much better* and *accessing it might be easier for everyone” *Midwife, FGD1.

Other participants expressed their need for locally appropriate clinical decision support tools and emphasized their wish to have a version of the SDA available in the local language ‘Kinyarwanda’.

*“I would like to add that it would be great to have this application in Kinyarwanda like I saw it having other language versions including Swahili, Hindi…. I guess this is a process, but we will be grateful if this is expedited. That will really be helpful. Thanks” *Nurse, FGD2.

In addition, nurses and midwives requested additional animated videos to be added to the SDA. These include; videos on antenatal care in line with the new WHO antenatal care models with a minimum of eight contacts as well as guidance on the identification of risk factors for birth complications before delivery.

*‘‘I think this application can be very helpful if they add some videos on antenatal care or how one could identify a woman in labor who would probably get a complication” *Midwife, FGD2.

Also important was the suggestion that the SDA application could have a discussion forum window or platform for the users across different hospitals and countries to share experiences about managing obstetric emergencies.

*“I wish that this application can have a kind of worldwide discussion forum where we can put comments and share experiences and views. For example, a midwife from X hospital in Rwanda can discuss and share her experience with midwives in other hospitals across multiple countries…Sharing like this can upgrade levels of knowledge for everyone. I think this would be very beneficial” *Midwife, FGD1.

### Interviews with key stakeholders

The analysis revealed two main themes: (1) benefits of the SDA as perceived by the key informants, and (2) future use of the SDA. These identified themes are shown in Fig. 1. The details on the main themes and sub-themes are presented, and are illustrated by quotations from the six KIIs.

#### Benefits of the SDA as perceived by the key informants

The key informants described the SDA as a useful tool and recommended its implementation in routine practices, specifically for the purpose of continuous learning without interrupting health services. Such benefit was depicted as fitting for the work environment in the context of Rwanda where numbers of healthcare providers were insufficient. Clinical staff require periodic training to stay on track with the knowledge and skills necessary for their practice. For their annual practice licence renewal, they need evidence of continuous professional development in addition to corresponding academic credits. SDA was therefore seen as a valuable solution as it offers on-the-job learning opportunities, assessment strategies, and possibilities of getting certificates.

*“This application is useful as it allows on-job continuous learning. We have been facing challenges for getting continuous learning opportunities for all staffs, now the SDA comes with this advantage. I recommend that it should be incorporated in routine practices” *Respondent, KII 3.

While the SDA was recommended for staff, because it captures the essential topics for BEmONC, key informants felt that this should in addition to the other sources of information including formal books and Ministry of Health guidelines that gives a broader view of obstetric care.

*“The SDA is good particularly for BEmONC but the SDA alone cannot solve all maternity care problems. Our staff need to use this application together with other sources of reliable information like the guidelines from the Ministry of Health” *Respondent, KII 1.

Another benefit highlighted was that the SDA is applicable in clinical practices by providing guidance on essential procedures that are needed to save maternal and newborn lives.

*“…We need safety in all we do, so this application is welcomed. I was happy to note that it provides concise information that will help nurses and midwives to take good decisions when dealing with childbirth complications” *Respondent, KII 6.

#### Future use of the SDA

 When KII participants were asked their opinions about the SDA cost, scalability, and sustainability, opinions on the future use of the SDA in Rwanda were apparent, along with the aspect of technology application in healthcare and its support in continuous learning. Participants perceived the estimated cost of piloting the SDA as affordable compared to physical training opportunities or online learning resources. They recommended the SDA implementation in consideration of its benefits, particularly the mLearning opportunity.

*“The cost is reasonable compared to other online learning tools…Also physical training has a double cost of time and resources. This application can train many people at the same time depending on individuals’ needs…I think this mLearning approach should be promoted in Rwanda as the country is looking to making healthcare services electronic-based…This application will be one of the solutions” *Respondent, KII 5.

One participant was concerned about the technology cost. But, he indicated that this cost could be alleviated by the fact that many healthcare providers own a smartphone. He suggested that they could use their own smartphone for future use of the SDA.

*“The technology cost is a bit high but as many people have smartphones and access to good internet, this might not be a problem. They could download the SDA on their own smartphones” *Respondent, KII 6.

In addition, participants expressed their wish to see the SDA implemented at a large scale, in all hospitals in Rwanda. However, they recommended that the SDA scalability needs to be preceded by a proper assessment and approvals by all required bodies.

*“Technology is in everything we do in healthcare and this application is useful…For future implementation, you need more sensitization and to involve all key stakeholders like MOH (Ministry of Health), RAM (Rwanda association of midwives), association of gynecologists and pediatricians…” *Respondent, KII 2.

*“I think after in-depth assessment and approval by MOH (Ministry of Health), the whole country (all hospitals) could use this application and it could help us to promote continuous learning while at work” *Respondent, KII 3.

Another participant highlighted the need for possible monitoring and follow-up programs to evaluate if the acquired knowledge and skills are translated into real clinical practices. He also suggested that the monitoring programs could be connected with the existing annual performance contracts and evaluation.

*“…Some mechanisms of monitoring could be put in place to assess if what has been learnt in the SDA is being practiced…Why not link this monitoring with imihigo (performance contracts)? People should think about it” *Respondent, KII 4.

Then again, participants indicated that the SDA could benefit a large variety of healthcare cadres like medical doctors.

*“…Not only nurses and midwives would benefit from the application, medical doctors can use it …Everyone needs to know how it works and its importance. *Respondent, KII 2.

Moreover, KII participants perceived the SDA as an easy to use tool with well explained topics. They indicated that the SDA could be sustained because it supports healthcare providers to manage childbirth complications. They also acknowledged the fact that the SDA provides useful information needed in routine clinical practices.

*“I think this application will be sustained…When you see the topics and how they are explained…This information is what our staff need every day for managing birth complications” *Respondent, KII 1.

Furthermore, participants suggested additional topics other than topics of BEmONC that could be added to the SDA to make it more useful and sustainable.

*“We have seen some additional topic like COVID 19 and infection prevention in this application. Other topics that are generally relevant for healthcare providers could be added…I am thinking of topics like: medical bio safety and bio security, patient centered care and quality improvement, ethics in clinical practice…all these topics could be tied to maternity care” *Respondent, KII 3.

## Discussion

The present study findings indicate that the SDA was acceptable to both the end-users and the key informants. End-users, nurses and midwives, used the SDA frequently, and viewed it as a useful tool that contributes to their professional growth and improve their practice. They frequently used it for education to revise and improve knowledge and skills, and as a decision support tool when immediate guidance was required to manage birth-related complications. Users felt that the SDA has improved their confidence and ability to manage childbirth complications, which they were previously insecure about. These findings are supported by a pre-post intervention study conducted with the same population, which found that the SDA in Rwanda led to a significant increase in nurses and midwives’ knowledge and skills scores for PPH and NR management six months after SDA introduction [19]. Other researchers similarly indicated that the SDA, when used as a training tool, has shown significant increases in knowledge and skills of different healthcare professionals [29, 30].

The perceived usefulness of the SDA seems to be related to its functionalities when applied to the clinical context of low resource settings [11]. This study emphasized the fact that the SDA offers continuous training opportunity without interrupting health services which is very beneficial due to the shortage of staffs. These findings mirror those of other studies that mHealth applications are increasingly being used in LMICs to improve health professionals’ access to evidenced and up to date information and training. In maternal and newborn healthcare services, mHealth application are being used as both training tools [31] and decision support tools [32]. The adoption of these applications in resources poor settings depend on their functions that need to be tailored to contexts of interest [33]. Other studies have similarly shown that mLearning are potential solutions to support continuous learning among health professionals in LMICs [34, 35].

Also, in the current study, the SDA was reported to be practical by helping nurses and midwives translate theoretical knowledge into practice with the support of visual instructions in the animated videos. Then again, with mLearning, the user has the opportunity to study/read multiple times and master the subject. A study in Kenya compared hands-on and video training for PPH management and reported that video training appeared to be as effective as hands-on training [36]. Thus, mHealth applications providing visual instructions via animated videos such as the SDA are potential solutions to improve knowledge and skills of nurses and midwives.

In addition, nurses and midwives had a positive experience with the SDA, they felt confident and better at their job when using the SDA, and reported it as an easy to use, intuitive and empowering tool. Participants were motivated to use the SDA in providing better services and suggested additional features and functions. These findings are in agreement with other studies which reported that the use of mHealth application is perceived as an opportunity for improving the quality of healthcare services with an effect on staff confidence [37], motivation [38], and self-efficacy [39]. Likewise, a realistic review about mHealth and the performance of maternal health care workers in low- and middle-income countries, revealed that health workers can be empowered to adopt and utilize mHealth in contexts where it is aligned to their needs, workload, training, and skills [40]. The review also noted that mHealth can empower health workers with skills and confidence when it is perceived as useful and easy to use [40]. Consequently, the SDA like other mHealth applications is considered an empowering tool that has a positive impact on healthcare delivery processes.

Moreover, in this study, the SDA was highly acceptable by nurses and midwives who admitted to continue using the SDA in their daily clinical practices. This level of user acceptance combined with the documented impact of the SDA is interesting considering the existing gap of ensuring access and adherence to BEmONC evidence-based clinical guidelines in low- and middle-income countries. Several studies have found a low adherence to evidence-based standards for maternal and newborn care particularly in low resource settings [41, 42]. Other studies have suggested the use of mHealth to improve the access and adherence to evidence-based guidelines [32, 43, 44]. Therefore, mHealth tools such as the SDA, which ensures access to the most recent BEmONC evidence-based clinical guidelines, and is demonstrably well accepted by its end-users, may help to improve the adoption of guidelines. The advantages of the SDA are that it is self-explanatory, available in many languages, is open source and free for download, and, once installed on the smartphone, does not require internet to function.

Furthermore, the SDA was appreciated by key informants in the management position at the district hospital level. On the other hand, those key stakeholders acknowledged the benefits of the SDA and were willing to advocate for its scalability for future use in Rwanda. They noted that the cost of piloting the SDA in two district hospitals was reasonable and predicted the possible sustainability of the SDA. However, they recommended a large scale research on the SDA and involvement of the required bodies. Likewise, Eze et al. [45] used a stakeholder perspective to analyze existing research on the mHealth process in developing countries, and reported that interactions between system developers and stakeholder groups are important for sustainability of mHealth [45]. Another research documented that in order to realize the potential benefits of mHealth, it is important to identify the relevant stakeholders and how they might be affected [46]. Other studies similarly found that the complexity of introducing mHealth into health care calls for strategies encouraging collaboration between multiple stakeholders to enhance successful implementation [47–50].

## Implications for research and clinical practice

The implications of this study are that the SDA was accepted by both end-users and key stakeholders due to its functions tailored to the Rwandan context. Among the beneficial functionalities of the SDA, there is the capacity of the SDA to function as a training tool and a decision support tool. This increases the confidence and the ability of nurses and midwives in providing quality care during obstetric and neonatal emergencies as well as improved routine obstetric and newborn care. The acceptability and the positive perceptions of the SDA in two district hospitals reflect a possible adoption of the SDA even on a large scale in Rwanda like in other low-resources settings. Evolving efforts for quality maternal and newborn care in Rwanda and similar contexts should consider the integration of SDA as an approach for training and as a clinical decision support tool for BEmONC in order to reduce maternal and newborn mortality. Future work could consider large scale studies of the SDA with maternal and newborn outcomes as primary outcomes.

## Strength and limitations of the study

The study is limited by the small sample size, which reflects the fact that the SDA was implemented only in two district hospitals due to funding limitations. Therefore, the findings of the current study cannot be generalized. Nevertheless, the evidence that the SDA was acceptable and perceived useful by both end-users and key stakeholders at the piloting settings sets the stage for expanding coverage and further testing of the SDA intervention to other hospitals in Rwanda. Additionally, the accuracy of our findings is increased by supporting the acceptability survey with focus group discussions to provide context to and clarify reasons behind some participant responses.

## Conclusions

The nurses and midwives perceived the SDA as an easy to use, useful, intuitive and empowering tool, which helps them update and revise their knowledge and skills, improves their confidence and makes them feel better to deliver quality obstetric care. Key stakeholders also perceived the SDA as a relevant and useful tool with a reasonable cost and recommended its implementation in routine practices. Overall, both end-users and key informants think that the SDA is beneficial and could be sustained. End-users suggested additional features that would improve the usability of the SDA. These included: a computer/ laptop version of the SDA, a SDA version in Kinyarwanda language, some additional videos, and a SDA discussion forum. Key informants also suggested additional topics that could be added to the SDA such as; medical bio-safety and bio-security, patient centered care and quality improvement, and ethics in clinical practice. The positive perceptions of the SDA in Rwanda is an indication that similar mLearning and mHealth decision making tools could be adopted as a means to improve knowledge and skills of nurses and midwives. The mHealth applications like the SDA have the potential to improve patient care, as they avail evidence-based clinical guidelines and can be easily adapted to contextual needs. Thus, credible, evidence-based, affordable mHealth applications like the SDA are needed to support clinical decision making and provide a platform for updates and continuous learning for nurses and midwives in Rwanda and other limited resource settings. The present study also adds to existing evidence about the benefits of mHealth applications in assuring quality BEmONC.

## Supplementary Information

**Additional file 1.**

**Additional file 2.**

**Additional file 3.**

## Data Availability

The datasets generated for this study will be made available from the corresponding author on a reasonable request.

## Abbreviations

BEmONC: Basic Emergency Obstetric and Newborn Care
FGD: Focus Group Discussion
KII: Key informant interview
mHealth: mobile health
mLearning: mobile learning
LMICs: Low- and middle-income countries
NR: Neonatal Resuscitation
PPH: Post-Partum Hemorrhage
RAM: Rwanda Association of midwives
UNICEF: United Nations International Children’s Emergency Fund
UNFPA: United Nations Population Fund
WHO: World Health Organization
