# Supplementary material for: A mixed-method study exploring experiences, perceptions, and acceptability of using a safe delivery mHealth application in two district hospitals in Rwanda
Source: BMC Nurs. 2022 Jul 4;21:176. doi: 10.1186/s12912-022-00951-w (PMC9251926; doi:10.1186/s12912-022-00951-w)
Supplement: Supplementary file 1 — Additional file 1. [file 12912_2022_951_MOESM1_ESM.pdf]

MATERNITY foundation

SAFE DELIVERY APP - ACCEPTABILITY SURVEY

You are about to answer questions regarding your experience of the Safe Delivery App. Please be as honest as possible.

Background variables

1. At which Hospital do you work?

a. Nyamata District Hospital

b. Masaka District Hospital

2. What is the highest level of education you have attained?

a. A0 Midwife b. A1 Midwife c. A0 Nurse

d. A1 Nurse e. A2 Nurse f. Other _____________

Questions regarding use of the Safe Delivery App

3. If you think about the last week, how many times did you use the Safe Delivery App?

a. I didn't use it at all b. I used it 1-3 times

c. I used it 4-6 times d. I used it almost every day

4. Which of the following Safe Delivery App features have you looked at?

a. Videos b. Action cards c. Drug list d. Procedures

5. In which of the following situations do you most often use the Safe Delivery App? Please select only one.

a. To revise my knowledge
b. During an emergency
c. In a normal work situation
d. To discuss with co-workers
e. To train clinical staff or assistants
f. To inform and guide women
g. Other

6. How do you most often access the Safe Delivery App? Please select only one option.

a. On my own phone b. The phone provided by the researcher c. Both

7. With who do you most often use the Safe Delivery App? Please select only one option.

a. By myself b. With a co-worker

8. **Where have you used the Safe Delivery App? Select all the locations that apply.**
   a. At work   b. At home   c. Other: _______________

9. **How much of the equipment and/or drugs seen in the Safe Delivery App do you have access to?**
   a. All   b. Most   c. Little   d. None

10. **How was your experience using the smartphone?**
    a. Very easy   b. Somewhat easy   c. Somewhat difficult   d. Very difficult

11. **The SDA is easy to use:**

Strongly disagree ☐   Disagree ☐   Undecided ☐   Agree ☐   Strongly Agree ☐

12. **Which feature do you appreciate the most? Please select only one.** a. Action cards
    b. Videos
    c. Drug list
    d. Procedures
    e. My Learning

13. **Do you think that the Safe Delivery App will be useful for you?**

    Strongly disagree ☐   Disagree ☐   Undecided ☐   Agree ☐   Strongly Agree ☐

14. **Which video is most useful for you? Select the one video that you find most useful.** a. Infection prevention
    b. Post Abortion Care (PAC)
    c. Active Management of Third Stage Labour (AMTSL)
    d. Hypertension
    e. Prolonged labour
    f. Post-partum Haemorrhage (PPH)
    g. Maternal Sepsis
    h. Manual Removal of Placenta
    i. Neonatal Resuscitation
    j. Newborn Management

15. Do you use the Safe Delivery App when you see the notifications? [Notifications are short reminder messages sent weekly by the App.]

a. Yes, most times b. Yes, sometimes c. No d. I did not see notifications

16. Have you logged in to My Learning?

a. Yes b. No

17. *If you have logged onto 'My Learning'* - Do you think My Learning will be useful for you?

Strongly disagree ☐ Disagree ☐ Undecided ☐ Agree ☐ Strongly Agree ☐

18. What do you like least about the Safe Delivery App? Select the one option that fits you best.

a. I do not understand the instructions.
b. I think it is difficult to navigate the smartphone.
c. The app does not function correctly (technical issues).
d. The app does not improve my practical skills.
e. The app does not add to my existing knowledge.
d. None applicable.

19. In your opinion, what is the most important quality of the app? Select the one option that fits you best.

a. I have become better at doing my job.
b. I can advise my patients better.
c. I feel more confident at work.
d. Fewer emergencies
e. Fewer mortalities/fatal cases
f. I did not notice any change.

20. Please write down one word that best describes the app.

____

Thank you for answering this questionnaire regarding your experience of the Safe Delivery App.
