# Supplementary material for: A mixed-method study exploring experiences, perceptions, and acceptability of using a safe delivery mHealth application in two district hospitals in Rwanda
Source: BMC Nurs. 2022 Jul 4;21:176. doi: 10.1186/s12912-022-00951-w (PMC9251926; doi:10.1186/s12912-022-00951-w)
Supplement: Supplementary file 2 — Additional file 2. [file 12912_2022_951_MOESM2_ESM.pdf]

# Appendix 1: Focus Group Discussion Guide

## A. Focus Group Discussion Preparation Checklist

The following preparations should be completed and materials obtained before each FGD:

- 12 copies of study information sheets
- 12 copies of consent forms
- 12 copies of audio-recording consent forms
- Box of pens
- Digital audio-recording equipment (tested for working condition)
- Backup batteries for audio recorder(s)
- Notebooks for facilitator
- Private room with at least 15 seats and enough space to arrange seats in a (semi)circle
- Food and drinks for participants

Name and Signature of Study Staff: ______________________Date: ____________________

## B. Checklist for Facilitator and Note taker

The FGD will only progress once the following are confirmed:

- All study consent forms have been signed and copies were given to participants
- All participants have signed the audio-recording consent form
- At least 6 participants in the group
- No more than 12 participants in the group

Note: Participants without the appropriate consent/assent forms or not meeting the inclusion criteria will be excluded.

Name and Signature of Study Staff: _______________________Date: __________________

## C. The questions for Focus group discussions

**Exploring the experiences, perceptions, and acceptability of the safe delivery mHealth application (SDA) among nurses and midwives in district hospitals in Rwanda**

1. Share with me your experience in delivering BEmONC before and after the use of the SDA? Probe for:
    a. The motivation for delivering BEmONC
    b. Challenges you face when delivering BEmONC
    c. Added value (if any) of using the SDA

2. After using the SDA, would you accept it in your routine practices? Probe for:

    a. Does the SDA meet your approval?
    b. Is the SDA appealing to you?
    c. Is the SDA useful?
    d. Is the SDA fitting your current work environment?
    e. Do you like the SDA?
    f. Are you welcoming the SDA?

3. After using the SDA, what do you think about its implementation in your daily practices? Probe for:
    a. Is the SDA implementable?
    b. Is the SDA possible in your daily practices?
    c. Is the SDA doable?
    d. Is the SDA easy to use?
4. After getting aware of the SDA and its roles and functionalities, what do you think about its sustainability (once implemented in your hospital)? Probe for:
    a. Do you intend to continue using the SDA in the future?
    b. Is the SDA durable?
    c. Can the SDA be maintained?
5. If you think that the SDA is not really fitting your current work environment, what would you need to change for it to be more acceptable?

**End of Interview, Thank you.**

# Appendix 2: Key Informant Interview Guide

### A. Key Informant Interview Preparation Checklist

The following preparations should be completed before each KII:

- 1 extra copy of study information sheets
- 1 extra copy of consent form
- 1 copy of audio-recording consent form
- Digital audio-recording equipment (tested for working condition)
- Backup batteries for audio recorder(s)
- Notebooks for interviewer
- Private room
- Food and drinks for interviewee

Name and Signature of Study Staff: _______________________ Date: __________________

### B. Checklist for Facilitator and Note taker

The KII shall only progress once the following are confirmed:

- Study consent form has been signed and copy were given to interviewee
- Interviewee has signed the audio-recording consent form

Name and Signature of Study Staff: ____________________Date: ____________________

### C. The questions for Key informant interviews

**Exploring the perceptions and acceptability of the safe delivery mHealth application(SDA) among key stakeholders (key informants in the management position at the district hospital level). They included maternity matrons, responsible for maternal and child health, and district hospital managers.**

The interview will start with an introduction of the SDA to the stakeholder. Explain its uses demonstrate how it functions and its implementation cost. After the introduction of the SDA, invite the participant to ask questions and comment on it. Once their concerns are addressed, explore the participants' perceptions and acceptability of the SDA.

1. Do you think nurses and midwives could benefit from the introduction of the SDA in district hospitals in Rwanda?
    a. Potential enablers
    b. Possible barriers
    c. Recommendations

2. After getting aware of the SDA and its roles and functionalities, what do you think about the implementation of the SDA in clinical practice? Probe for:

a. Does the SDA meet your approval?
b. Is the SDA appealing to you?
c. Do you like the SDA?
d. Are you welcoming the SDA?
e. Is the SDA useful?

3. After getting aware of the SDA and its roles and functionalities, what do you think about the use of the SDA in clinical practices at district hospitals of Rwanda? Probe for:
   a. Does the SDA seem implementable?
   b. Is the SDA possible?
   c. Is the SDA doable?
   d. Is the SDA easy to use?

4. After getting aware of the SDA pilot costs in two district hospitals, do you think that it is affordable to the Rwandan context? Probe for:
   a. Technology cost
   b. Training cost
   c. Intervention cost
   d. Total cost

5. After getting aware of the SDA and its roles and functionalities, what do you think about its sustainability (once implemented in district hospitals in Rwanda)? Probe for:
   a. Can nurses and midwives continue to use the SDA in the future?
   b. Is the SDA durable?

**End of Interview, Thank you**
