# Supplementary material for: A mixed-method study exploring experiences, perceptions, and acceptability of using a safe delivery mHealth application in two district hospitals in Rwanda
Source: BMC Nurs. 2022 Jul 4;21:176. doi: 10.1186/s12912-022-00951-w (PMC9251926; doi:10.1186/s12912-022-00951-w)
Supplement: Supplementary file 3 — Additional file 3. [file 12912_2022_951_MOESM3_ESM.pdf]

Consolidated criteria for reporting qualitative research (COREQ) checklist

Adapted from: Tong A, Sainsbury P, Craig J. Consolidated criteria for reporting qualitative research (COREQ): a 32-item checklist for interviews and focus groups. Int J Qual Heal Care. 2007;19(6):349–57.

Title of the manuscript: A mixed-method study exploring experiences, perceptions, and acceptability of using a Safe Delivery mHealth Application in two district hospitals in Rwanda.

Number and Item	Guide questions/responses	Section and page # where reported
**Domain 1: Research team and reflexivity**		
Personal Characteristics		
1. Interviewer	*Which author/s conducted the interviews?*<br>Interviews were conducted by AN and one research assistant (OT) assisted in taking notes and supervising the tape recorder (see Acknowledgments).	Methods- Data collection and analysis - Qualitative data, paragraph 4
2. Credentials	*What were the researcher's credentials?*<br>Aurore Nishimwe, PhD student<br>Daphney Nozizwe Conco, PhD<br>Marc Nyssen, PhD<br>Latifat Ibisomi, PhD	Title page
3. Occupation	*What was their occupation at the time of the study?*<br>AN was a PhD student and DNC, MN and LI were supervisors.	N/A
4. Gender	*Were the researchers male or female?*<br>Both genders were represented.	N/A
5. Experience and training	*What experience or training did the researcher have?*<br>AN had training in qualitative research methods during her PhD courses. DNC, MN and LI are conversant with qualitative research methods and have published research studies using qualitative methods.	N/A
Relationship with participants		
6. Relationship established	*Was a relationship established prior to study commencement?*<br>No prior relationship was established between interviewers and participants.	N/A
7. Participant knowledge of the interviewer	*What did the participants know about the researcher?*<br>Reasons for conducting the study and institutional affiliations of all researchers were stated in the Participant Information Sheet and Consent Form.	N/A
8. Interviewer characteristics	*What characteristics were reported about the interviewer?*<br>No interviewer characteristics were reported to participants.	N/A
**Domain 2: study design**		
Theoretical framework		

9. Methodological orientation and theory	*What methodological orientation was stated to underpin the study?*<br>The thematic analysis with an hybrid approach was utilized.	Methods- Data collection and analysis – Qualitative data ,paragraph 4
Participant selection		
10. Sampling	*How were participants selected?*<br>Purposive sampling was utilized.	Methods- Data collection and analysis- Qualitative data, paragraph 3,4
11. Method of approach	*How were participants approached?*<br>Selected nurses, midwives, and key stakeholders were approached face-to-face.	Methods- study participants, paragraph 1
12. Sample size	*How many participants were in the study?*<br>24 participants in the FGDs and six participants in the KIIs	Methods- study participants, paragraph 1
13. Non-participation	*How many people refused to participate or dropped out?*<br>No individuals refused to participate or dropped out of the study.	N/A
Setting		
14. Setting of data collection	*Where was the data collected?*<br>All interviews were conducted at the district hospitals	Methods- Qualitative data, paragraph 4
15. Presence of non-participants	*Was anyone else present besides the participants and researchers?*<br>No, only the participant and interviewers were present. Interviews were held in private rooms at district hospitals.	Methods- Data collection and analysis- Qualitative data, paragraph 4
16. Description of sample	*What are the important characteristics of the sample?*<br>Participants were nurses and midwives with a work experience over 6 months in obstetric care and full-time employed in the selected district hospitals. The study also included key stakeholders (key informants in the management position at the district hospital level). They included maternity matrons, responsible for maternal and child health, and district hospital managers.	Methods-study participants, paragraph 1
Data collection		
17. Interview guide	*Were questions, prompts, guides provided by the authors? Was it pilot tested?*<br>The interview guide was developed by the authors and prompts were given during interviews, if needed. Two pilot interviews were conducted.	Methods- Data collection and analysis – Qualitative data, paragraph 4
18. Repeat interviews	*Were repeat interviews carried out?*<br>No repeat interviews were conducted.	N/A
19. Audio/visual recording	*Did the research use audio or visual recording to collect the data?*	Methods- Data collection and

	All interviews were audio-recorded, with participants' informed consent.	analysis – Qualitative data, paragraph 4
20. Field notes	*Were field notes made during and/or after the interview?*<br>Field notes were made to record additional information, as necessary.	Methods- Data collection and analysis – Qualitative data, paragraph 4
21. Duration	*What was the duration of the interviews?*<br>60 and 90 minutes for FGDs, and 30 and 45 minutes for KIIs	Methods- Data collection and analysis – Qualitative data, paragraph 4
22. Data saturation	*Was data saturation discussed?*<br>Yes, participants were recruited until thematic saturation was achieved.	Methods- Data collection and analysis – Qualitative data, paragraph 4
23. Transcripts returned	*Were transcripts returned to participants for comment and/or correction?*<br>No	N/A
**Domain 3: analysis and findings**		
Data analysis		
24. Number of data coders	*How many data coders coded the data?*<br>Two interviews were double-coded by AN and DC. AN coded all remaining interviews.	Methods- Data collection and analysis – Qualitative data, paragraph 4
25. Description of coding tree	*Did authors provide a description of the coding tree?*<br>Codes represented distinct viewpoints on each theme and subtheme.	N/A
26. Derivation of themes	*Were themes identified in advance or derived from the data?*<br>Themes were derived from the data collected.	Methods- Data collection and analysis – Qualitative data, paragraph 4
27. Software	*What software, if applicable, was used to manage the data?*<br>Nvivo 11 Plus software was used to assist in coding.	Methods- Data collection and analysis – Qualitative data, paragraph 4
28. Participant checking	*Did participants provide feedback on the findings?*<br>Participants did not provide feedback on the findings; however, findings will be shared with participants upon publication.	N/A
Reporting		
29. Quotations presented	*Were participant quotations presented to illustrate the themes findings? Was each quotation identified?*	Results, all paragraphs

	Yes, participant quotations were identified by age and duration experience in obstetric care.	
30. Data and findings consistent	*Was there consistency between the data presented and the findings?*<br>Yes	Discussion, paragraphs 1-7
31. Clarity of major themes	*Were major themes clearly presented in the findings?*<br>Major themes resulting from the interviews are listed in figure 1 : Thematic framework.	Results section
32. Clarity of minor themes	*Is there a description of diverse cases or discussion of minor themes?*<br>Yes, sub-themes were discussed.	Results section
